# Supplementary material for: Improved calculation method for dry modal analysis of four-stage centrifugal-pump rotor system based on concentrated-mass method
Source: PLoS One. 2024 Jun 28;19(6):e0306061. doi: 10.1371/journal.pone.0306061 (PMC11213302; doi:10.1371/journal.pone.0306061)
Supplement: S1 File — (PDF) [file pone.0306061.s002.pdf]

Calculation of natural frequency and vibration mode

Four-stage centrifugal pump with balance disc：

```
A=[0.000000795424*5.61,0.00000141047*5.266,0.0000014534*5.266,0.000001081
98*5.25,0.000000491344*6.6;0.00000141047*5.61,0.00000285066*5.266,0.0000031
0759*5.266,0.00000236382*5.25,0.00000108244*6.6;0.0000014534*5.61,0.0000031
0759*5.266,0.00000376108*5.266,0.00000302593*5.25,0.00000141447*6.6;0.00000
108198*5.61,0.00000236382*5.266,0.00000302593*5.266,0.00000270519*5.25,0.00
000133563*6.6;0.000000491344*5.61,0.00000108244*5.266,0.00000141447*5.266,
0.00000133563*5.25,0.000000745039*6.6]
vpa(A)
[V, D]=eig(A)
vpa(D)
```

```
A=[0.000000795424*5.19/1.136,0.00000141047*5.266/1.136,0.0000014534*5.266/1.
136,0.00000108198*5.25/1.136,0.000000491344*6.198/1.136;0.00000141047*5.19/
1.256,0.00000285066*5.266/1.256,0.00000310759*5.266/1.256,0.00000236382*5.25
/1.256,0.00000108244*6.198/1.256;0.0000014534*5.19/1.256,0.00000310759*5.266
/1.256,0.00000376108*5.266/1.256,0.00000302593*5.25/1.256,0.00000141447*6.19
8/1.256;0.00000108198*5.19/1.265,0.00000236382*5.266/1.265,0.00000302593*5.2
66/1.265,0.00000270519*5.25/1.265,0.00000133563*6.198/1.265;0.000000491344*
5.19/1.187,0.00000108244*5.266/1.187,0.00000141447*5.266/1.187,0.00000133563
*5.25/1.187,0.000000745039*6.198/1.187]
vpa(A)
[V,D]=eig(A)
vpa(D)
```

Four-stage centrifugal pump with back blade：

```
A=[0.00000154123*5.997,0.00000216732*5.479,0.00000192654*5.479,0.00000110
776*5.997;0.00000216736*5.997,0.00000346777*5.479,0.00000327512*5.479,0.000
00192654*5.997;0.00000192654*5.997,0.00000327512*5.479,0.00000346777*5.479,
0.00000216736*5.997;0.00000110776*5.997,0.00000192654*5.479,0.00000216736*
5.479,0.00000154123*5.997]
vpa(A)
[V,D]=eig(A)
vpa(D)
```

```
A=[0.00000154123*5.479/1.106,0.00000216732*5.479/1.106,0.00000192654*5.479/
1.106,0.00000110776*5.479/1.106;0.00000216736*5.479/1.236,0.00000346777*5.47
9/1.236,0.00000327512*5.479/1.236,0.00000192654*5.479/1.236;0.00000192654*5.
479/1.236,0.00000327512*5.479/1.236,0.00000346777*5.479/1.236,0.00000216736*
5.479/1.236;0.00000110776*5.479/1.106,0.00000192654*5.479/1.106,0.0000021673
6*5.479/1.106,0.00000154123*5.479/1.106]
vpa(A)
[V,D]=eig(A)
vpa(D)
```
