# Supplementary material for: Improved calculation method for dry modal analysis of four-stage centrifugal-pump rotor system based on concentrated-mass method
Source: PLoS One. 2024 Jun 28;19(6):e0306061. doi: 10.1371/journal.pone.0306061 (PMC11213302; doi:10.1371/journal.pone.0306061)
Supplement: Attachment — Submitted filename: Response to Reviewers.doc.docx [file pone.0306061.s003.docx]

**Responses to Reviewers’ Comments**

Thank you for your valuable comments and suggestions regarding our manuscript. We have carefully considered each of the comment and performed the necessary revisions to improve the quality and clarity of our manuscript. These changes are indicated in red in the revised manuscript. Our responses to the reviewers’ comments are provided below:

**Academic editor:**

1.Abstract should be improved to avoid repetition of text.

Response: Thank you for your suggestion. We have revised the Abstract to eliminate this repetition. The revised version succinctly summarizes the research purpose, content, methodology, and conclusions and highlights the contributions and innovations of our study.

2.Contours in Figures 3 and 6 are not of good quality. The values on color map are also not clear.

Response: Thank you for highlighting this. We have considered your feedback in revising the manuscript. Specifically, we have improved the image quality by saving them in .tif format directly using the ANSYS software. This resulted in higher-quality images and clearer contours. Additionally, we have ensured that the values on the color map are clearly visible. These changes have been implemented in the revised map file.

3.The comparison with experimental studies should be included in the revised paper.

Response: Thank you for your suggestion. We appreciate your recommendation to include comparisons with experimental studies in the revised manuscript. However, we would like to emphasize that our focus is to propose an optimized mathematical model. In this study, we compared the results before and after optimization with ANSYS simulation results. We determined the effectiveness of the optimization model based on the reduction in the error values. Furthermore, we validated the applicability of the optimized model to a centrifugal pump rotor system with back blades. We believe that considering the factors affecting the errors in our study is reasonable. Our study can be used to improve the accuracy of analytical methods. Moreover, the ideas that you proposed provide directions for our future investigations.

4.The differences with the previous similar papers (Refs. 11 and 29) of the author need to discussed in detail.

Response: Thank you for your comment. Owing to the increased number of references, references 11 and 29 were updated to 31 and 32, respectively. The relevant content is briefly summarized in the Literature Review section of the manuscript. Reference [31] focused on a double-support four-stage centrifugal pump rotor system, where a simplified “four-segment four-concentration” mathematical model and an ANSYS model were used to solve the mechanical model of a D-type balanced-mass four-stage centrifugal pump rotor system. An improved mathematical model combining “five-segment four-concentration” and modal resistance-bending stiffness correction factors was proposed, and the effect of impeller-mass eccentricity on the modes was analyzed. The authors of [32] investigated a cantilever four-stage centrifugal pump rotor system using a simplified lumped-mass mathematical model and ANSYS to solve the natural frequencies of the mass-balanced rotor system. An optimized mathematical model integrating shaft system mass and modal stiffness correction factors was proposed, and the effect of impeller-mass eccentricity on the modes was analyzed. These two studies, as well as our current study, employed different structures of four-stage centrifugal pump rotor systems; hence, different mathematical models were established. Additionally, the factors considered during the optimization of the models varied owing to differences in the fixed methods and structures of the pump. Factors such as the unit-size division of the geometric model, material type, stiffness, and mass distribution contributed to significant errors in the natural frequencies. The focus of this study is the selection of lumped masses and the section moment of inertia. Based on the considerations above, an optimized mathematical model was established. The Literature Review section briefly describes this, with the modifications indicated in red.

**Reviewer #1:**

1-The authors should be followed the instruction of the journal in all parts and sections in this manuscript.

Response: Thank you for the comment. We acknowledge the importance of adhering to the instructions provided by the journal throughout the manuscript. We have carefully reviewed your comments and ensured that all the contents of the manuscript comply with the journal’s guidelines. The revised contents are indicated in red.

2-Complete mathematic calculation model with all nomenclature missing. Please check the number of each section, equation, and chart.

Response: Thank you for your comment. We have thoroughly reviewed the manuscript. We have revised the images to ensure clarity, renamed some of the image titles, and added references to the equations. All the changes are highlighted in red in the manuscript.

3-The abstract needs more quantitative results. The abstract section is an important and powerful representation of the research. It is better that the results should be presented with the support of specified data. Please provide your contribution and work novelty.

Response: Thank you for the suggestion. We have modified the abstract accordingly to highlight the key contributions of our study. In particular, we have summarized the research content, where we emphasized the use of an analytical method based on concentrated mass and the comparison of results with ANSYS simulation results. We presented specific numerical values to demonstrate the significant reduction in errors after optimization. The errors were within 5% in general, which satisfies the operational requirements and validates the feasibility of the optimization method. The focus of the study was the selection of concentrated mass and the moment of inertia of cross-sections, from which we derived an optimized mathematical model. Furthermore, we validated the applicability of the optimization method to another rotor system, which enabled the assessment of the system's vibration characteristics under specific operating conditions, thereby providing important insights for pump design and optimization. The rotor system with a balance disc fixed by a simply supported beam used in this study has not been reported previously. The modified sections of the Abstract are highlighted in red.

4-The authors should indicate this technique to enhance system performance. Also, the author should add more references that discuss the effect of using this technique. It is recommended that the authors carry out wide analysis and comparison with the state-of-the-art studies.

Response: Thank you for your comment. We have revised the Literature Review section to include more references. Additionally, we have discussed the application of the lumped-mass method in modal analysis, the effects of different structural materials on modal behavior, and the use of modal analysis in structural damage detection. The added information highlight the importance of modal analysis and its significance in optimizing the structural design of centrifugal pumps, improving pump performance and efficiency, reducing noise and vibration, and ensuring safe equipment operation. Changes to the manuscript are highlighted in red.

5-Most tables and figures are needed improve the quality of all tables and figures.

Response: Thank you for your comment regarding the quality of the tables and graphics in the manuscript. We have scrutinized every table and graph and improved their quality accordingly. Specifically, we improved the resolution of the image, adjusted its file format to enhance clarity, ensured that all labels and annotations were accurately matched, and renamed some images. These improvements have been incorporated into the revised manuscript, along with updated figure numbers.

6-Add references for all equations.

Response: Thank you for your comment. We have added references to all the equations in the manuscript. The equations were derived primarily from References [34] and [35], and the added citations are indicated in red.

7-I would also expect to validate with two more experimental works available in the literature.

Response: Thank you for your comment. We agree that additional studies will help us better understand the details of these interactions and enhancements. However, we would like to emphasize that our primary focus is to propose an optimized mathematical model to improve the accuracy of the concentrated-mass method. By comparing the values obtained from the analytical calculations with those from the ANSYS simulation, the significant reduction in errors validates the effectiveness of the optimized model. Thus, the proposed approach can facilitate future investigations.

8-The literature review must be improved. Please highlight in the literature review the differences between previous papers and your paper. Please clearly indicate the knowledge gap and prove that it is a really not analyzed area of the field. Please indicate new approach / new methods in a comparison to the existing investigations (literature review should be extended and add below references).

Response: Thank you for your comment. We have revised the Literature Review section as follows:

(1) We provided an overview of studies pertaining to the modal characteristics of rotor systems conducted by scholars both domestically and abroad.

(2) We discussed the application of modal-analysis methods in structural damage detection.

(3) We elaborated on the effects of different structural materials on the modes.

(4) We highlighted studies by foreign scholars pertaining to the application of the centralized-mass method used in this study to other fields.

By performing a literature review, we identified the cutting-edge research directions and application areas of modal analysis. Currently, studies regarding the design optimization of mathematical models for centrifugal-pump rotor systems are limited. This paper delves into this aspect to improve the accuracy of modal calculations and provide a foundation for the structural design of centrifugal pumps and their vibration and noise reduction. Additionally, we have included the references you mentioned in the revised manuscript (see References [3], [4], [5], [10], [11], [16], [17], and [42]). Modifications to the literature review and references are highlighted in red.

9-You need to add error analysis of your results and add the error bars in your graphs to indicate your accuracy measurements.

Response: Thank you for your comment. We have added two error-comparison diagrams to the revised manuscript, as shown below.

Comparison of analytical calculations and optimization results

Based on the error comparison presented, we clearly observed a significant decrease in the optimized error values. The error-comparison charts clearly demonstrated the effectiveness of the optimized mathematical model in improving the calculation accuracy of the analytical method.

Comparison between analytical calculation and optimization results of back-vane centrifugal pump

Based on a comparison between the analytical calculation and optimization results, the errors in the analytical calculation values decreased from 13.11%, 12.85%, 9.91%, and 7.2% to 3.7%, 3.86%, 0.57%, and 2.87%, respectively, thus indicating the effectiveness of the optimized mathematical model.

10-Improve work justification. Also, add more analysis about velocity and pressure contours.

Response: Thank you for your comment. We understand the importance of velocity and pressure contours in certain scenarios. However, our primary focus is to propose an optimized mathematical model to improve the accuracy of the concentrated-mass method. This involves the application of both the concentrated-mass analytical method and ANSYS simulation, through which the natural frequencies and mode shapes can be determined. Based on the numerical results, we analyzed the desired modes and deformations. Additionally, we prioritized modal analysis as it aligns with our research objectives and investigation scope. We discovered that the velocity cloud diagram can show the velocity distribution of a fluid flowing through a pipeline or device, whereas the pressure cloud diagram can show the pressure level of a fluid at different positions. However, our study is based on the dry mode; hence, the above-mentioned diagrams are not relevant. Nevertheless, we will ensure that our study is fully demonstrated and will consider providing additional analyses of velocity and pressure contours in our future fluid–solid coupled studies.

11-More quantitative conclusions should be presented. Please prepare additional comparisons, some percentage differences. There is a lack of quantitative conclusions which should contain main findings from the paper and highlight the new and high novelty and contribution of your work to the field.

Response: Thank you for your comment. We have revised the Conclusion section accordingly. Specifically, we have succinctly summarized the main contents of our paper and outlined the key conclusions of our study. By performing numerical comparisons, we demonstrated a significant reduction in errors, i.e., errors within 5% in general after optimization. The errors are primarily due to the improper selection of concentrated-mass segments and the incomplete consideration of the cross-sectional moment of inertia factors. Based on these findings, we derived correction formulas for the optimized mathematical model. Furthermore, we validated the feasibility of the optimization model. The proposed optimization model is the focus and novelty of our study. Enhancing the accuracy of the concentrated-mass method facilitates the structural design of centrifugal pumps by avoiding noise and vibration. This is important for the stable operation of centrifugal pumps and cost reduction. Modifications to the conclusions are indicated in red.

12-Present the mathematical equation of the boundary conditions and initial condition.

Response: Thank you for your comment. The mathematical model used in this study applies the equation of motion of a multi-degree-of-freedom linear structural system as follows:

Modal analysis considers the inherent characteristics of the system; therefore, undamped free vibration is analyzed, i.e.,$C=0,\left\{F(t)\right\}=0$. The free-vibration equation of the system can be expressed as:

To solve the mode in ANSYS, one must establish the model, partition the grid, apply constraint conditions, and finally solve the output results of the model. Grid partitioning significantly affects the results of modal analysis. The finer the grid, the more accurate are the calculation results. In this study, the entire model was selected for geometric size adjustment. The unit size was 7 mm, and the entire model was segmented into 183431 nodes and 102154 units. A constraint condition was applied to simulate a simply supported beam; one end was the fixed hinge support, and the other end was the sliding hinge support. The left end of the simply supported beam was selected to constrain the displacements in the X- and Z-directions, whereas the right end was selected to constrain the displacement in the Z-direction. The entire beam was selected to constrain the displacement in the X-direction.

13- I would also suggest including in the conclusion section but also in several other places in the manuscript discussion and comparison with findings from other authors with similar published research work.

Response: Thank you for your comment. The manuscript has been revised to include a brief comparison of two articles by the same authors in the Literature Review section. Reference [31] focuses on a double-supported four-stage centrifugal pump rotor system, where a simplified “four-segment four-concentration” model and an ANSYS model were used to solve the mechanical model of a D-type balanced-mass four-stage centrifugal pump rotor system. Additionally, an improved mathematical model combining “five-segment four-concentration” and a modal resistance-bending stiffness-correction coefficient was proposed, and the effect of impeller-mass eccentricity on mode was analyzed. The authors of [32] investigated a cantilevered four-stage centrifugal pump rotor system using a simplified concentrated-mass mathematical model and ANSYS to solve the natural frequency of the rotor system under mass balance. They proposed an optimized mathematical model that integrated the shaft system mass and modal stiffness correction coefficient, and then analyzed the effect of impeller-mass eccentricity on mode. These two studies, in addition to our current study, examined the different structures of four-stage centrifugal pump rotor systems. Because of the differences in the pump structures, the mathematical models used and the factors considered during optimization varied. Factors such as the geometric model unit-size division, material type, stiffness, and mass distribution resulted in significant errors in the natural frequencies. The focus of our study is to consider the selection of concentrated mass and the cross-sectional moment of inertia to derive an optimized mathematical model. This has been briefly described in the Literature Review section and is highlighted in red.

14- The conclusion section on lacks in summative conclusions. The main results, novelty and academic contributions should be emphasized in this section. Moreover, are the results obtained in this paper really applicable in other similar researches?

Response: Thank you for your comment. As per your suggestion, we have revised the Conclusion section. In the first paragraph of the Conclusion, we have added a statement to provide an overview of the main results. Additionally, we have organized the conclusions into bullet points for a clearer summary. To validate the effectiveness of the optimized model, we introduced another centrifugal-pump rotor system with balanced axial action: a four-stage centrifugal pump with back blades. The same ISG100-65-200 water pump impeller was used, along the concentrated-mass method and an optimized mathematical-model formula. The errors in the natural frequencies decreased from 13.11%, 12.85%, 9.91%, and 7.2% to 3.7%, 3.86%, 0.57%, and 2.87%, respectively, all of which were within the 5% error range. This validated the applicability of the optimized model to four-stage centrifugal pumps with balanced discs. Modifications to the Conclusion section are highlighted in red.

15- In the discussion development, it is very important to emphasize points of agreement or disagreement between results in this work and others cited in references part of manuscript.

Response: Thank you for your comment. As per your feedback, we have revised the manuscript accordingly. Specifically, in the Literature Review section of the manuscript, we highlighted the points of agreement or disagreement between the results obtained in this study and those cited in the references. Additionally, we discussed two relevant papers by the same authors.

16- Authors should discuss limitations of the current study and possible improvements for future directions/research works. Authors are requested to check the reference format and correct some inconsistent formats.

Response: Thank you for your insightful comment. We appreciate your suggestion and have revised the manuscript accordingly. Specifically, we have expanded the Introduction section to discuss the applicability of our research methods to other fields, as well as the effects of different structural materials on modal analysis. Furthermore, we have reviewed and corrected the citation-format inconsistencies throughout the manuscript. The modifications are highlighted in red.

17- Finally, I strongly recommend the author to read through the whole text and correct it to make it more reader-friendly.

Response: Thank you for your suggestion. We have carefully reviewed the entire manuscript and corrected it accordingly to enhance readability and provide clarity. The modifications are highlighted in red.

**Reviewer #2:**

1. Some variables need to be indicated in italics to avoid misunderstandings.

Response: Thank you for your comment. We have addressed this issue by italicizing the relevant variables in the manuscript. The modifications are highlighted in red.

2. The pictures in the article are blurry, a clearer picture will help to understand the contribution.

Response: Thank you for your comment. As per your suggestion, we have performed a few adjustments to enhance the image quality. Specifically, we improved the image clarity by adjusting the file format during the image-saving process. The figures presented in the revised manuscript are clearer and easier to interpret.

3. The author mention that this is an improved method, but just consider the division of the mass more carefully in the mass matrix and consider the stiffness of the impeller in the stiffness matrix after optimization. Calling it an improved method requires careful consideration.

Response: Thank you for your comment. Owing to the structural differences in centrifugal pumps, the natural frequencies and modes of the system are affected by their inherent characteristics, such as the elastic modulus and density of the materials, as well as the mass and stiffness of the system. Prior to deriving the optimized model, we performed extensive calculations, which indicated that factors such as the elastic modulus and density did not significantly affect the natural frequencies. The observed discrepancies are attributed to the following reasons:

(1) Repeated consideration of the axial spacing mass between impellers 1 and 2 and between impeller 4 and the balance disc.

(2) Non-consideration of the inertia moments at the impellers and balance disc when calculating the cross-sectional moment of inertia of the system.

The stiffness of the shaft section is determined not only by the bending stiffness of the pump shaft but also by the bending stiffness of the front and rear cover plates. Because the cross-sectional area of the blades is much smaller than those of the front and rear cover plates, only the stiffness coefficients at the front and rear cover plates are considered at the impeller. Hence, a correction factor α is introduced, which is related to the effective length of the rotor, the pump shaft diameter, the hub diameter, the hub thickness of the impeller, and the thickness of the front and rear cover plates.

$$\alpha=\frac{1}{\frac{T-t_{1}}{T}+\frac{t_{2}+t_{3}}{T}(\frac{d}{D})^{4}}$$

The mathematical model derived in this study is the novelty and focus of this study. Applying this optimized model to a four-stage centrifugal pump with back blades yielded favorable optimization results, thus further confirming the effectiveness of the model.

4. In this article, only Ansys result is used for verification, if a comparison with experimental result is made will become more convincing.

Response: Thank you for your comment. We acknowledge that additional experimental validation will enhance the persuasiveness and completeness of this study. The primary focus of our study is to propose an optimized mathematical model to improve the accuracy of the concentrated-mass method. The optimization effect of the analytical method requires a reference value. We selected the value obtained via ANSYS simulation as a control and evaluated the feasibility of the model based on the size of the errors. The value obtained experimentally is the same as that of obtained via ANSYS simulation; therefore, we will consider incorporating it in our future studies.

5. The derivation and analysis generally is lack of innovation and the form of computation result is too simple which should be significantly improved.

Response: Thank you for your comment. The most important aspect was the application of the following characteristic equation:

The vibration mode vector and natural frequency $\omega$ were analyzed. The centrifugal pump shaft used in this study was fixed by a simply supported beam, as shown in the following figure.

Schematic diagram of simply supported beam

Based on the mechanics of materials, the deflection of a beam is expressed as

$$v=\left\{\begin{matrix} -\frac{Pbx}{6EIl}(l^{2}-x^{2}-b^{2})(0\leq x\leq a) \\ -\frac{Pbx}{6EIl}\left[\frac{l}{b}\left(x-a\right)^{3}+(l^{2}-b^{2})x-x^{3}\right](a\leq x\leq l) \end{matrix}\right.$$

This equation is important for deriving the flexibility influence coefficient at the impeller and balance plate, which is the solution for$\delta_{ij}$ in the manuscript. The flexibility matrix of the system is expressed as

$$K^{-1}=\left[\begin{matrix} \delta_{11} & \delta_{12} & \delta_{13} & \delta_{14} & \delta_{15} \\ \delta_{21} & \delta_{22} & \delta_{23} & \delta_{24} & \delta_{25} \\ \delta_{31} & \delta_{32} & \delta_{33} & \delta_{34} & \delta_{35} \\ \delta_{41} & \delta_{42} & \delta_{43} & \delta_{44} & \delta_{45} \\ \delta_{51} & \delta_{52} & \delta_{53} & \delta_{54} & \delta_{55} \end{matrix}\right]$$

Here, the matrix M is composed of various concentrated mass segments. The process above is for solving the natural frequency and vibration mode using an analytical method. When deriving the optimized model, we considered the effects of material elastic modulus, density, system mass, and stiffness on the natural frequencies. After extensive calculations and repeated derivations, we considered the errors introduced by the selection of concentrated-mass segments and cross-sectional moments of inertia in the mathematical model calculations. Subsequently, correction coefficients were derived. These correction coefficients were related to the effective length of the rotor, the pump shaft diameter, the hub diameter, the hub thickness of the impeller, and the thickness of the front and rear cover plates of the impeller.

**Reviewer #3:**

1-In table 2, it’s hard to find the new nature frequency. It’s a simple copy of table 1.

Response: Thank you for your comment. Table 1 presents the theoretically calculated natural frequencies, whereas Table 2 provides a comparison between the theoretical calculations and simulation results, along with their respective errors. The largest error was 12.96%. This implies that the mathematical model of the lumped-mass method must be optimized.

2-What the key point for the authors to show the model shape again. I think the original, FEM and optimization system should have similar mode shape.

Response: Thank you for your comment. By comparing Figures 2(a)–2(e) and 5(a)–5(e), we observed that the modal vibration directions before and after optimization obtained from the theoretical calculations were consistent. This indicates that the derived optimization model minimally affects the modal shapes and thus the deformation of the centrifugal-pump rotor system in the radial direction. The variation in the modal shapes constitutes the modal analysis; therefore, the modal shapes before and after optimization were compared.

3-Usually, the first nature frequency should very close to the FEM and experiment results.

Response: Thank you for your comment. We conducted modal analysis using analytical methods and via ANSYS simulation. The first natural frequencies obtained from the analytical method and ANSYS simulation were 21.737 and 24.982 Hz, respectively. A concentrated-mass method was employed for vibration analysis, and no errors were generated during the calculation process. A three-dimensional model exported from UG was used in the ANSYS Workbench modal simulation, and the centrifugal-pump type and fixation method were consistent with those of the analytical method. The modal analysis yielded the natural frequencies of various modes. We observed a certain error in the first-order natural frequency obtained using these methods.

4-For a high-quality paper, I think a simple experimental verify is needed.

Response: Thank you for your comment. We understand the importance of experimental verification in ensuring the robustness of the research findings. The primary focus of our study is to propose an optimized mathematical model to improve the accuracy of the concentrated-mass method. The optimization effect of the analytical method requires a reference value. We selected the value obtained via ANSYS simulation as a control and evaluated the feasibility of the model based on the size of the errors. The value obtained experimentally was the same as that obtained via ANSYS simulation results; therefore, either one of the values can be selected. We appreciate your suggestion and will consider incorporating experimental validation in our future studies.

5-Novelty needs to be explicit in abstract.

Response: Thank you for your comment. We have revised the Abstract and emphasized the key contributions of our study. The novelty and focus of our study is the consideration of the selection of concentrated mass and cross-sectional moments of inertia. Based on this, we derived an optimized mathematical model and validated it using another rotor system to demonstrate its applicability. The vibration characteristics of the system under specific operating conditions can be assessed by analyzing the natural frequencies and mode shapes of a centrifugal pump, thereby providing important insights for pump design and optimization. Modifications to the abstract is highlighted in red.

6-Expand figure captions so figures are almost self-explanatory.

Response: Thank you for your suggestion. We have expanded the figure captions to provide more detailed explanations, ensured that the figures are almost self-explanatory, and modified the image to ensure that the values are clearly visible. The modifications are highlighted in red.

7-Broaden and update the literature review to better connect to the current effort in the field in the context of mechanical sciences; for papers like this one we expect no less than 40 journal papers including 15 recent ones to be critically discussed. Do not cite text books or manuals.

Response: Thank you for your comment. We have expanded and updated the Literature Review section to include a more comprehensive discussion of recent advancements in rotor-system modal characteristics, as well as the application of modal analysis in structural damage detection, the effects of different structural materials on modal behavior, and, most importantly, the broader applications of the concentrated-mass method utilized in this study. We have increased the number of references to 43, as per your suggestion, to ensure that the literature review focuses only on journal articles, excluding textbooks and manuals.

8-Conclusions should be stronger.

Response: Thank you for your comment. We have revised the Conclusion section as per your suggestion. We have provided a concise summary of the main research content and outlined the key conclusions of our study in bullet points. We demonstrated a significant reduction in errors, i.e., errors within 5% in general after optimization. The observed errors were attributed to the unreasonable selection of concentrated-mass segments and the incomplete consideration of factors related to the cross-sectional moment of inertia. Hence, we derived a correction formula for the optimized mathematical model. Finally, we validated the feasibility of the optimized model and emphasized its significance as the novelty of our study. The modifications are highlighted in red.
